# Glymphatic System and Psychiatric Disorders: A Rapid Comprehensive Scoping Review

**DOI:** 10.2174/1570159X22666240130091235

**Published:** 2024-01-31

**Authors:** Tommaso Barlattani, Paolo Grandinetti, Alexsander Di Cintio, Alessio Montemagno, Roberta Testa, Chiara D’Amelio, Luigi Olivieri, Carmine Tomasetti, Alessandro Rossi, Francesca Pacitti, Domenico De Berardis

**Affiliations:** 1 Department of Biotechnological and Applied Clinical Sciences (DISCAB), University of L’Aquila, Via Vetoio, Coppito, 67100 L’Aquila, Italy;; 2 National Health Service, Department of Mental Health, Psychiatric Service of Diagnosis and Treatment, Hospital G. Mazzini, ASL 4 Teramo, Italy

**Keywords:** Glymphatic system, aquaporin-4, astrocytes, psychiatric disorders, mood disorders, depression

## Abstract

**Background:**

Since discovering the glymphatic system, there has been a looming interest in exploring its relationship with psychiatric disorders. Recently, increasing evidence suggests an involvement of the glymphatic system in the pathophysiology of psychiatric disorders. However, clear data are still lacking. In this context, this rapid comprehensive PRISMA-ScR (Preferred Reporting Items for Systematic Reviews and Meta-Analyses extension for Scoping Reviews) scoping review aims to identify and analyze current evidence about the relation between the glymphatic system and psychiatric disorders.

**Methods:**

We conducted a comprehensive review of the literature and then proceeded to discuss the findings narratively. Tables were then constructed and articles were sorted according to authors, year, title, location of study, sample size, psychiatric disorder, the aim of the study, principal findings, implications.

**Results:**

Twenty papers were identified as eligible, among which 2 articles on Schizophrenia, 1 on Autism Spectrum Disorders, 2 on Depression, 1 on Depression and Trauma-related Disorders, 1 on Depression and Anxiety, 2 on Anxiety and Sleep Disorders, 8 on Sleep Disorders, 2 on Alcohol use disorder and 1 on Cocaine Use Disorder.

**Conclusion:**

This review suggests a correlation between the glymphatic system and several psychiatric disorders: Schizophrenia, Depression, Anxiety Disorders, Sleep Disorders, Alcohol Use Disorder, Cocaine Use Disorder, Trauma-Related Disorders, and Autism Spectrum Disorders. Impairment of the glymphatic system could play a role in Trauma-Related Disorders, Alcohol Use Disorders, Cocaine Use Disorders, Sleep Disorders, Depression, and Autism Spectrum Disorders. It is important to implement research on this topic and adopt standardized markers and radio diagnostic tools.

## 1 INTRODUCTION

**Fig. (1):**
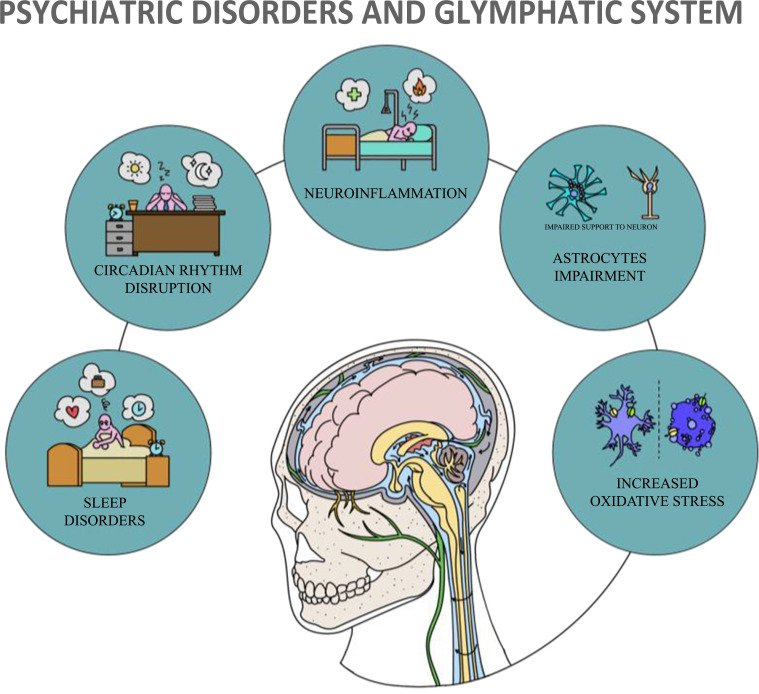
Common physiopathological trajectories between Psychiatric Disorders and Glymphatic System involvement. Adapted from Schubert *et al*. 2019.

In 2012, Iliff and *et al*. described for the first time the glymphatic system, a complex system located in the central nervous system (CNS) of mammals [1]. Due to the similarities with the peripheral lymphatic system, they coined the term glymphatic, referring to its two main components: the fluid and glial components, respectively represented by the subarachnoid cerebrospinal fluid (CSF), interstitial fluid (ISF) and astrocytes, and their interactions [2]. This unique network is composed of astroglial processes that create a layer surrounding all the arterioles, capillaries, and venules of the brain, forming perivascular tunnels [3-5]. The small perivascular spaces (PVSs) found between the processes and the vessel walls, through which ISF moves, provide CSF movement through the brain parenchyma, leading to osmotic clearance of metabolites [6, 7]. Specifically, regarding fluid movements, the CSF, produced mainly in the choroid plexus (CP), and the ISF, are drained into the so-called Virchow-Robin space (VRS), which is formed by the PVSs surrounding the superficial cerebral vessels and proximal penetrating vessels [8, 9]. From the VRS termination, CSF moves into the basal lamina surrounding the cerebral capillaries [10]. CSF and ISF end up draining from the CNS through a lymphatic pathway located in the dural meninges [11-15], as well as along nerve conduits in the cribriform plate, finally leading to the cervical lymphatic vessels [16] and ending into the superficial and deep cervical lymph nodes. The other main component of such systems is represented by astrocytes, which are essential components of the blood-brain barrier (BBB) that play a crucial role in the interaction between neurons and brain vessels [17]. Indeed, together with microglia, pericytes, endothelial cells and the basal lamina, astrocyte end-feet form the neurovascular unit (NVU) [4, 5]. In addition, astrocytic processes express aquaporin-4 (AQP-4), a channel that allows the exchange of water inside and outside the cell [18] and likely plays an important role in promoting the influx of perivascular CSF [19] into the brain parenchyma, where it mixes up with ISF [20]. Interestingly, the ultrastructure of astrocytic processes [21, 22] and AQP-4 channel expression appear to be regulated by a circadian rhythm [23]. Other essential functions of astrocytes are to remove brain waste compounds, to provide support and nutrients to neurons, being moreover involved in homeostatic processes, ions concentrations, repair mechanisms, synaptic regulatory systems [24-26] and regulation of cerebral blood flow around cerebral vessels [27, 28]. In summary, the glymphatic system allows CSF and ISF perfusion of the CNS and solute exchange between these two fluids, especially during sleep, and presumably under the influence of arterial pressure flow, vasomotion, and respiration [6, 7, 19, 29, 30]. Being the glymphatic system influenced by circadian regulation [31] and predominantly active during sleep, it has been hypothesized that an excess of catecholamines, especially prominent during wake time, may reduce the functioning of the glymphatic network [32, 33]. Astrocytes’ activity may also be regulated by the circadian rhythm, being adjacent to axons and neuron bodies during wakefulness, weaving smaller capillaries and leaving very little space between them [21, 22, 34]. In contrast, in deep physiological sleep, astrocytes contract, causing a significant increase in the space between compressed glial cells and neurons, allowing the CSF to flow intensely through brain tissue [21, 35, 36]. Therefore, the glymphatic system stands out as a critical tool for the elimination of macromolecule wastes, reactive oxygen species (ROS), cytokines, antigens, tau species, and amyloids [1, 2, 37] and the transport of solutes crucial for brain metabolism such as glucose [38], lipid signalling molecules [39], and apolipoprotein E [40]. Increasing evidence links dysfunction of the glymphatic system to CNS pathologies [41], as, for instance, malfunctioning of the glymphatic system has been highlighted in Parkinson's disease (PD) [42], Alzheimer's disease (AD) [43, 44] (Fig. **1**), and in Multiple Sclerosis (MS) [45]. Moreover, the comorbidity of these disorders with psychiatric illness [46] and the common physiopathological trajectories, including increased oxidative stress [47, 48], neuroinflammation [49, 50], alterations in sleep [51, 52], circadian rhythms disruption [53, 54], and astrocyte damage [55, 56] leads to the hypothesis that such system may also be involved in the pathogenesis of psychiatric disorders alone. It is, therefore, noteworthy that several recent studies are discussing the potential correlation and involvement of the glymphatic system in the context of mood disorders [57, 58]. However, data on glymphatic system dysfunction and correlation with psychiatric disorders are still scarce and solid evidence is lacking. In this context, the present study aims to highlight current evidence on the correlation between the glymphatic system and psychiatric disorders, the dysfunction of this system in the context of psychiatric disorders, and to fill the gaps in the literature on this topic.

## 2 METHODS

A comprehensive review of the literature was carried out on Pubmed up to February 26, 2023. Considering the extensive aim of the study, we used Medical Subject Headings (MeSH) descriptors (“Glymphatic System” [Mesh] AND “Psychiatric Disorders” [Mesh]). To maximize the sensitivity of our study, we did not provide additional terms besides “Glymphatic System” and “Psychiatric Disorders.” Subsequently, the studies included were discussed with a narrative overview. Aiming to cover a broad literature overview and considering that strong evidence regarding the glymphatic system and psychiatric disorders is still lacking, a rapid comprehensive PRISMA-ScR scoping review, following the statement guidelines for scoping reviews [59], was identified as the best method to carry out the present study [60]. Articles selected were published between August 2012, when the glymphatic system was first described and defined [1], and February 2023. Only articles regarding the glymphatic system and psychiatric disorders, assessing the correlation between the glymphatic system and psychiatric disorders, or changes in psychiatric disorders and glymphatic system in a longitudinal perspective were included; finally, we included other studies that may provide useful information on clinically observable psychiatric manifestations related with the glymphatic system. Given the breadth of the topic and the aim of the study, in the present review, we only selected studies that addressed psychiatric disorders about the glymphatic system as a network and not about any single components of the glymphatic system (*e.g*., AQP-4, Astrocytes, CP, PVSs, VRS, CSF, ISF) or providing indirect measures of the glymphatic system functioning. In this review, we will include under the term “psychiatric disorder” all major psychiatric diseases (*e.g*., mood disorder (bipolar disorder, depression), Schizophrenia (SCZ) and psychosis, personality disorders (PDs), trauma and post-traumatic stress disorder (PTSD), anxiety, obsessive-compulsive disorder (OCD), autism spectrum disorders (ASDs), attention deficit and hyperactivity disorder (ADHD), substance abuse disorders (SUDs) (APA, 2013). Given the importance of sleep disorders (SDs) in the context of psychiatric disorders and the unique bidirectional relation between SDs and psychiatric disorders [51], articles providing useful information on SDs and sleep alterations as a manifestation of psychiatric disorders were also included. Considering the limited number of studies on this topic, we have included both clinical and preclinical studies. We excluded studies in languages other than English. Being the present study is a scoping review, the quality of the studies has not been addressed during the selection process [61]; therefore, meta-analysis, reviews, and systematic reviews were excluded. Purely narrative papers, editorials, book chapters, letters to editors, comments, and case reports, with small samples (< 10 subjects), were excluded as they would not provide better insight into the researched topic. Two independent reviewers screened citations for inclusion. Data extraction was conducted by one reviewer and verified by a second reviewer. Tables were then constructed, and articles were sorted out by authors, year, title, location of study, sample size, psychiatric disorder, the aim of the study, principal findings, and implications. The main findings of the included studies have been summarized in Table **1**. Results were then discussed.

## 3 SEARCH RESULTS

The initial Pubmed search yielded a total of 212 results, while 3 additional titles were identified through other sources (website searching, citation tracking, and reference chaining). 3 records were excluded as not full text. During the screening process of the remaining 212 records, 154 were excluded as the article’s primary focus was not the correlation between the glymphatic system and psychiatric disorders, as their content was considered as irrelevant in the light of the present study's aim. Among the 58 remaining papers, 31 were excluded since they did not meet the above-listed inclusion criteria: 2 were systematic reviews, 23 were reviews, 2 were editorials, 1 was a study protocol, 1 was a viewpoint, and 2 were written in languages other than English, namely in Japanese and Russian. Of the final 27 eligible records, 7 were excluded as they did not provide adequate information about the relation between the glymphatic system and psychiatric disorders. 20 papers were finally identified as of particular interest. The selected articles are presented in Table **1** and discussed in the narrative overview. For specifics about the study design, consult Fig. (**2**).

## 4 NARRATIVE OVERVIEW

Among the 20 papers identified as suitable for this review, 9 were carried out in China, 5 in the United States (USA), 1 in China/USA, 1 in Italy, 1 in Norway, 1 in Australia, and 2 in Taiwan. 9 of the selected studies employed a murine sample, the others human samples. Regarding the psychiatric disorders taken into consideration, we found 2 articles focusing on SCZ, 1 on ASDs, 2 on depression, 1 on depression and trauma-related disorders, 1 on depression and anxiety, 2 on anxiety and SDs, 8 on SDs, 2 on Alcohol use disorder (AUD), and 1 on cocaine use disorder (CUD). Regarding the relationship between the glymphatic system and SCZ, Wu *et al*. conducted a case-control study in 2020 in Taiwan, recruiting 292 SCZ consecutive cases and 100 healthy controls (HC) of Southern Han Chinese descent [62]. For the association study, the 100 cases were matched for age, sex, and education to 100 of the HC. The aim was to investigate the relationship between AQP-4 gene polymorphisms and SCZ. In particular, three single nucleotide polymorphisms (SNPs) were found in SCZ patients belonging to the Southern Chinese Han population. When compared with HC, individuals with SCZ had a higher frequency of T alleles in rs1058424 and G alleles in rs3763043. In addition, an association between decreased risk of SCZ and AA genotype for both rs1058424 was found. The TCG haplotype was associated with a potential risk of developing SCZ, while the ACA haplotype was associated with a decreased risk and maintained statistical significance after Bonferroni correction. Therefore, this study was able to highlight an association between AQP-4 gene polymorphisms and SCZ in the Southern Han Chinese population. Wu *et al*. in 2018 carried out another study in Taiwan recruiting 190 patients diagnosed with SCZ of Southern Han Chinese descent [63]. The aim of the study was to investigate whether the gene polymorphisms and haplotype of AQP-4 influenced serum S100 calcium-binding protein B (S100B) level and clinical symptoms in patients with SCZ. Results showed that patients with the TAA haplotype of the AQP-4 polymorphism were likely to present increased serum S100B levels, as well as negative symptoms, and poor control of neuroinflammation. A logS100B level > 1.78 was moreover supposed to be sufficiently specific to predict a higher severity of negative symptoms. Therefore, results suggest that AQP-4 influences brain neuroinflammation in individuals with SCZ given its important role in maintaining BBB integrity, structure, and permeability. This study also provides a possible association between the involvement of genetic variations in the AQP-4 gene and the functional outcome of patients with SCZ. We found only one study addressing ASDs. This work, conducted in China in 2022 by Li *et al*., compared 30 children diagnosed with ASDs and 25 HC through diffusion tensor imaging (DTI) along the perivascular space (DTI-ALPS) [64]. The authors observed that patients with ASDs presented a significantly lower ALPS index than HC, suggesting that subjects had glymphatic dysfunction in patients with ASDs. This study underlines the importance of the DTI-ALPS approach in assessing the function of the glymphatic system in ASDs. Two studies included used a murine model to assess the role of the glymphatic system in the pathogenesis of depression. One was conducted in China in 2020 by Liu *et al*., using a sample of adult male mice, with the aim of testing the prediction that chronic unpredictable mild stress (CUMS) manifests with disturbed glymphatic function and whether dietary supplementation with polyunsaturated fatty acids (PUFAs) can ameliorate these deficits, alleviating the cognitive decline associated with depression [65]. The authors formulated also a secondary hypothesis: that dietary PUFA supplementation, but not escitalopram (ES), would restore glymphatic function and relieve depression-like behaviours and cognitive impairments in CUMS mice. Adult male C57BL/6 mice undergoing an eight-week CUMS paradigm were therefore treated either with PUFA or with ES. Results showed that significant depression-like behavioural symptoms and cognitive dysfunction in CUMS mice are accompanied by marked impairment of the glymphatic system. Even though the depression-like behavioural symptoms are relieved by treatment with the serotonin selective reuptake inhibitor (escitalopram), cognitive impairments in CUMS mice persist, unless the underlying disruption of the glymphatic system is restored. Daily oral supplementation with PUFA restores glymphatic system functions in CUMS mice and, therefore, relieves depression-like symptoms and cognitive deficits. Male mice were also studied by Xia *et al*. in 2017, in a work conducted in China, with the aim of identifying the pathogenetic relationship between depressive disorder and AD through a chronic unpredictable mild stress (CUMS) model, while assessing the function of the glymphatic pathway using fluorescence tracers [66]. The accumulation of endogenous mouse and exogenous human amyloid beta 42 (Aβ42) in CUMS-treated mice, with or without treatment with the antidepressant fluoxetine, was evaluated. Xia *et al*. observed that CUMS reduces AQP-4 expression in the cortex and hippocampus, and hypothesized that this may explain the altered glymphatic clearance, and the subsequent accumulation of Aβ42 in the brain, thus demonstrating that the dysfunction of the glymphatic system after injury by depressive disorder is a critical element in the development of AD, and the antidepressant fluoxetine may reverse this pathogenesis under chronic stress. The therapeutic effect of fluoxetine has also been associated with increased regulation of AQP-4 expression in astrocytes. However, while treatment with fluoxetine alone was effective in chronic stress, it had no effect in control mice. Ranti *et al*., in a work conducted in the USA in 2022, focused on the link between the glymphatic system’s neuroanatomy *via* PVS and trauma experience in patients with major depressive disorder (MDD), compared with HC, using 7-Tesla MRI and a semi-automated segmentation algorithm [67]. For this study, they recruited 21 patients with major depressive disorder (MDD) and 27 healthy controls (HC). Results yielded showed that the number of traumatic events correlated with the total PVS volume, both in patients with MDD and in the general population. Such results are suggestive not only of a relationship between glymphatic dysfunction, especially related to BBB integrity and psychological trauma but also that glymphatic impairment may play a role in trauma-related symptomatology. After sorting for age and sex, the number of traumatic events, specifically those eliciting fear, helplessness, or horror, was found to be positively correlated with total PVS volume both in the population with MDD and in the overall study population. These results suggest that PVS could potentially be a useful biomarker for estimating symptom severity and its long-term impact on neuroanatomical structures in the context of psychological trauma and MDD. Chen *et al*., in a 2021 cross-sectional study from China, recruited 431 patients with PD from Beijing Tiantan Hospital from May 2016 to August 2019, with the aim to explore the multi-dimensional effects of cerebral small vessel disease (CSVD) on PD outcomes, including depression and anxiety [68]. Patients with idiopathic PD underwent complete magnetic resonance imaging (MRI) examination necessary for evaluating CSVD and fulfilled, among others, Hamilton Anxiety Rating Scale (HAMA) and Hamilton Depression Rating Scale (HAMD) questionnaires. Significant associations were observed between the CSVD burden and HAMA score, as well as between the CSVD burden and HAMD score in multivariable model 2, while only a tendency toward a significant association was found in model 1. The study then concluded the total CSVD burden was significantly associated with depression/anxiety in patients with PD. Two studies deepened the relationship between the glymphatic system, SDs, and anxiety using a murine model. In particular, Liu *et al*., in a study conducted in China in 2017, tested the effect of continuous theta burst stimulation (cTBS) on a mouse sleep deprivation model [69]. The researchers studied the effect of cTBS on glymphatic pathway clearance in normal and in sleep- deprivation C57BL/6J mice, detecting AQP-4 polarization by immunofluorescence, and assessing anxiety-like behaviors trough open field (OF) tests. Sleep deprivation seems to reduce influx efficiency along the PVS, disturb AQP-4 polarization and induce anxiety-like behaviors. cTBS significantly attenuated the decrease in efficiency of solute clearance usually incurred with sleep deprivation, restored the loss of AQP-4 polarization and improved anxiety-like behavior in sleep-deprived animals. Results implied that cTBS had potential protective action against neuronal dysfunction, including anxiety-like behavior induced by SDs. Vasciaveo *et al*. in a work conducted in Italy in 2023, analyzed the effect of sleep fragmentation in wild-type and 5xFAD mouse models [70]. The OF tests were used to assess anxiety‐like behavior and spontaneous motor activity in all groups. Fisher’s multiple comparison tests revealed increased anxiety in fragmented (F) 5xFAD mice by reducing the time spent in the open arms, when compared to not fragmented (NF) 5xFAD and F-wt mice, which in contrast spent more time in the open arms compared to their control group (NF-wt). Sleep fragmentation induces a general acceleration of AD progression in 5xFAD mice, while in wild-type mice it affects cognitive behaviors in particular learning and memory. Results from this study, therefore, demonstrate how AQP-4 modulation is a crucial player of the glymphatic system activity, and sleep fragmentation differentially affects AQP-4 expression, and such influence varies according to the stage of the disease, where an up-regulation has been observed in younger animals, while such change could not be detected in older ones. Among the selected studies, 8 focused on the relation between glymphatic alterations and SDs. 2 of such studies employed a murine model, while 6 human samples. Achariyar *et al*. carried out a study in 2016 in the USA employing a mouse model in order to establish whether apoE in CSF, secreted by the CP, is homogeneously distributed into the brain and whether this distribution pattern was altered by sleep deprivation [40]. Results highlighted the contribution of glymphatic fluid as a transporting system involved in the delivery of CP/CSF-derived human apoE to neurons and how sleep deprivation suppresses glymphatic CSF-derived apoE distribution and clearance into the brain. To better delineate the role of AQP-4-mediated glymphatic clearance impairments in the brain following chronic sleep insufficiency, Zhang *et al*., in a work conducted in the USA and China in 2020, investigated glymphatic transport and accumulation of Aβ and Tau proteins following 7 days of sleep disruption [71]. They then proceeded to assess the pathophysiological consequences of AQP-4 deletion in this process using a sample of adult AQP-4-null mice and a control group of wild‐type (WT) mice. AQP-4 deletion resulted in an impairment of glymphatic transport and accumulation of β‐amyloid and Tau proteins in the brain following sleep disruption. AQP-4-null sleep-deprived mice, when compared with WT‐sleep-deprived mice, presented severe activation of microglia, neuroinflammation, synaptic protein loss in the hippocampus, as well as decreased working memory. AQP-4‐mediated glymphatic clearance ameliorates brain impairments caused by abnormal accumulation of metabolic wastes following chronic sleep deprivation. Thus, it could be considered a potential target for sleep‐related disorders. Siow *et al*. conducted a cross-sectional study recruiting 84 participants aged 60 or older in China in 2022 to explore the associations between human glymphatic function, sleep, neuropsychological performance, and cerebral gray matter volumes [72]. N2 phase sleep duration and the apnea-hypopnea index were independently associated with DTI-ALPS. Higher DTI-ALPS was associated with better Everyday Cognition (ECog) language scores and better Consortium to Establish a Registry for Alzheimer's Disease Neuropsychological Battery (CERAD-NB) word list learning delayed recall subtest scores after covarying for age and education. Higher DTI-ALPS was also associated with higher gray matter volume after controlling for normal variations due to age, sex, and total intracranial volume. Significant associations were identified between glymphatic function and sleep, underlying the importance of sleep for brain health. This study also revealed associations between DTI-ALPS, neuropsychological performance, and cerebral gray matter volumes, suggesting the potential of DTI-ALPS as a biomarker for cognitive disorders. To study whether MRI-visible enlarged PVS could be useful neuroimaging markers to predict cognitive impairment in chronic insomnia patients, Wang *et al*. in China in 2022, compared 156 patients with chronic insomnia and 79 age-matched healthy individuals [73]. Reported results demonstrated MRI-visible enlarged PVS in the frontal cortex, centrum semiovale, basal ganglia, and hippocampus of chronic insomnia patients with impaired cognition (ICG when compared with normal cognition (NCG) patients. The increased MRI-visible enlarged PVS in the frontal cortex, centrum semiovale, and basal ganglia were also associated with the increased CSF Aβ42, t-tau, and p-tau levels in ICG patients. MRI-visible enlarged PVS in the basal ganglia, and centrum semiovale had high sensitivity and specificity in distinguishing ICG chronic insomnia patients from the NCG group. In conclusion, MRI-visible enlarged PVS in the basal ganglia and centrum semiovale might be valuable imaging markers to predict cognitive impairment in chronic insomnia patients. To explore the role of AQP-4 in AD, Rainey-Smith *et al*. investigated in a study conducted in 2018 in Australia, genetic variation across the AQP-4 gene with a particular focus on the relationship with, and between, sleep quality and quantity and brain Aβ burden [74]. This cross-sectional study recruited 462 participants aged 60 years or older, among which 222 also underwent Αβ imaging performed *via* positron emission tomography (PET) and genetic data were derived from a genome-wide SNPs array. Sleep quality was assessed with the Pittsburg Sleep Quality Inventory (PSQI). One AQP-4 variant, rs72878776, was associated with poorer overall sleep quality, while several SNPs moderated the effect of sleep latency (rs491148, rs9951307, rs7135406, rs3875089, rs151246) and duration (rs72878776, rs491148 and rs2339214) on brain Aβ-amyloid burden. This study demonstrated how genetic variation of AQP-4 is crucial in the relationship between self-reported sleep quality and brain Aβ burden as assessed by PET in cognitively normal older adults. The aim of the study conducted by Piantino *et al*. in 2021 in the USA was to explore the relationship between the number of mild traumatic brain injuries (mTBIs), poor sleep, and PVS burden in a cohort of Iraq/Afghanistan Army veterans after adjustment for clinically relevant covariates, as well as to establish the correlation between PVS burden and persistent postconcussive symptoms in this cohort [75]. To achieve such goals, the authors used a validated and automated image segmentation tool to quantify the number, volume, morphology, and location of PVS in the whole brain. An analysis of MRI data was then performed, and sleep quality was assessed by means of the PSQI. The number of mTBIs sustained in the military is associated with PVS burden, and this relationship is modulated by poor sleep. In the selected cohort, increased PVS burden is associated with worse post concussive symptoms, especially poor balance. Thus, quality of sleep modulates the relationship between the number of mTBIs sustained in the military and PVS volume. In persons with poor sleep, the increase in PVS volume observed with each subsequent mTBI is higher than in those who sleep well. Shokri-Kojori *et al*. in a work conducted in the USA in 2018, tested 20 healthy controls after a night of rested sleep, defined as rested wakefulness (RW) [76]. The aim of the study was to test whether one night of sleep deprivation would increase ABB in the hippocampus, which shows some of the earliest structural and functional changes in AD. The second objective of the study was to test the association between a history of poor sleep and higher ABB in the hippocampus, precuneus, and medial prefrontal cortex. One night of sleep deprivation, relative to baseline, resulted in a significant increase in Aβ burden in the right hippocampus and thalamus. These increases were associated with mood worsening following sleep deprivation, although they were not related to the genetic risk (APOE genotype) for AD. Eide *et al*. in a work conducted in Norway in 2020, compared a cohort of individuals undergoing one night’s total sleep deprivation with a cohort allowed unrestricted sleep and further followed both groups the following 24 h of unrestricted sleep to examine whether CSF tracer enhancement in human parasagittal dura is affected by sleep deprivation [77]. After one night of sleep deprivation (at 24 h), there was neither evidence for altered tracer enrichment in the parasagittal dura nor after a day of unrestricted sleep (at 48 h). The hypothesis of altered molecular egress to parasagittal dura after sleep deprivation was therefore not supported by this study. One of the selected studies, carried out by Chen *et al*. in China in 2020, focused on CUD [78]. Using a mouse model of noncontingent cocaine exposure, the research group evaluated alteration in glymphatic function, including CSF-ISF exchange and solute clearance from the brain, during repeated cocaine exposure and after withdrawal. This study found that the glymphatic system is consistent impairments caused by cocaine use, observable even after a seven-day withdrawal period. Such changes may be due to the deceleration and reduction of cerebral blood flow, decrease of the cerebral arterial wall pulsatility, astrogliosis, and the loss of AQP-4 polarity. These results suggest a multimodal mechanism through which cocaine impairs the glymphatic function, which may contribute to the development of neurocognitive disorders in patients with cocaine addiction. Our review included 2 papers studying the correlation between the glymphatic system and AUD, both conducted employing mice samples. Specifically, Lundgaard *et al*., in the USA in 2018, studied the effects of acute and chronic ethanol exposure and withdrawal from chronic ethanol exposure on glymphatic function [79]. In particular, they investigated glymphatic function in awake mice that had undergone acute and chronic ethanol exposure or chronic exposure followed by 24 hours of withdrawal. Low doses of chronic ethanol intake were associated with a significant decrease in GFAP expression, with little change in the cytokine profile, when compared with the saline group. These observations suggest that ethanol has a J-shaped effect on the glymphatic system, whereby low doses of ethanol increase glymphatic function. Conversely, chronic ethanol intake induced reactive gliosis and perturbed glymphatic function, which may contribute to and higher risk of developing dementia. Interestingly, this study found that the suppression of glymphatic function was not permanent and could be restored at 24 h after termination of chronic binge alcohol administration. The other study was conducted in China in 2020 by Liu *et al*., with the aim of investigating the exact mechanisms by which acute and chronic alcohol exposure impairs glymphatic function in the brain [80]. The glymphatic function seems to be impaired by acute and chronic alcohol application through different mechanisms. Acute alcohol intake reduces the influx and efflux function of the glymphatic system, possibly through stimulating the release of β-endorphin (β-EP) by and decreasing cerebrovascular pulsatility, while chronic alcohol consumption leads to activation of astrocytes and a widespread loss of perivascular AQP-4 polarization in the brain, resulting in an irreversible impairment of the glymphatic function. The reduction in the glymphatic function and CSF-ISF exchange may decrease the efficiency in the clearance of interstitial solutes, which could lead to solute accumulation such as Aβ deposition in the brain, thus contributing to the development of cognitive decline and dementia in chronic alcohol abusers. Conversely, this study found that the loss of perivascular AQP-4 polarization in the chronic binge-level alcohol-treated animals persisted even after 2 weeks of withdrawal.

## 5 DISCUSSION

This study aims to provide an overview of currently available studies investigating the relationship between the glymphatic system and psychiatric disorders. Regarding clinical studies, our review showed how the glymphatic system plays a role in SCZ, depression, anxiety disorders, SDs, AUD, trauma-related disorders, and ASD. Regarding preclinical studies, our work showed how the glymphatic system is involved in AUD, CUD, SDs, anxiety disorders and depression. Taken together, results observed in our review revealed how the glymphatic system plays a role in several psychiatric disorders, such as SCZ, depression, anxiety disorders, SDs, AUD, CUD, trauma-related disorders, and ASDs [33, 40, 62, 63, 65-79, 81]. Instead, in relation to other disorders (ADHD, OCD, bipolar disorder, PDs, and PTSD), no scientific evidence is currently available. Some of the considered studies suggested alteration of the glymphatic system in the context of some psychiatric disorders. Specifically, our findings suggest the involvement of glymphatic system impairment in trauma-related disorders, AUD, ASD, CUD, SDs, and depression [33, 65-67, 69, 70, 72, 78, 79, 81]. There are some considerations that must be made to discuss our results. Psychiatric disorders often present alterations in circadian rhythms [54], sleep alterations [51], increased oxidative stress [48], neuroinflammation [49], alterations in monoamines [82] and impaired glutamate secretion [83], and microglial and astrocytic damage [55, 84]. All the above-mentioned alterations could be correlated with, and possibly compromised by, dysfunctions of a single component of the glymphatic system but also related to impairments of the system itself [58, 85]. Hence, our study confirms the role of AQP-4 as a key component of the glymphatic system and its potential role as a marker of glymphatic impairment, also in the context of psychiatric disorders [62, 63, 70, 71, 74, 86, 87]. Furthermore, our study sheds light on the possible reliability of DTI-ALPS employment to assess glymphatic function [72, 81, 88]. Moreover, the role of TBI is a risk factor for psychiatric disorders in relation to glymphatic alterations [75, 89, 90]. Results from our review also confirm the close interrelationship between psychiatric and neurologic disorders, which could be quite likely mediated by the glymphatic system, confirming, for instance, the risk of dementia developing in the context of a psychiatric disorder [33, 46, 65, 66, 68, 72, 78, 79, 91]. The reported involvement of SDs [85] in the impairment of the glymphatic system confirms the role of this cross-system in the context of psychiatric illness, given the central role of SDs in the development of psychiatric diseases [51, 92-94], possibly through alterations in the glymphatic system itself, as it has already been hypothesized for mood disorders [95]. Specifically, when approaching depression under the glymphatic system perspective, data from our review seem consistent with the hypothesis that cognitive alterations found in depression could be considered as a prodrome of dementia itself [96]. Such thesis is supported by findings of markers of astroglial damage, such as S100 β and glial fibrillar acid protein (GFAP), along with alterations in AQP-4 expression [97, 98] and with alterations in choroid plexus (CP) morphology [99] found both in dementia and in depressed patients presenting cognitive alteration [100-102]. In addition, our study supports the role that restoration of the gut microbiome axis and supplementation of nutrients, such as PUFAs and Omega-3, may play in alleviating symptoms of depression [103] and modulating the glymphatic system [71]. Regarding anxiety disorders, similar considerations to those previously made for depression can be made, especially regarding the role of AQP-4 [104], astrocytes [105] and neuroinflammation [106]. Available studies on SCZ suggest that increased S100 β levels in CSF and serum may be found in subjects with early-onset SCZ [107], while astrocyte damage could play a role in the development of the disorder [108]. Regarding CUD and AUD, there is consistency in the literature regarding the involvement of AQP-4, along with astrogliosis and neuroinflammation, in addictive behavior [109-111]. Our data on ASDs seem compatible with other studies, including those revealing increased extra-axial CSF volume in children with normal-to-high risk of manifesting ASDs [112], as well as astrocyte involvement, as demonstrated by the presence of markers of astrocyte damage, and AQP-4 in the brains of ASDs subjects [113]. In addition, although no clarifying data on PTSD were found, findings on glymphatic system alteration in trauma-related disorders and glymphatic system-related sleep alterations in a population at risk for PTSD, such as veterans, suggest the need for further investigation. Supporting such hypothesis, some data report indirect measures of glymphatic system impairment in PTSD [81] and alterations in norepinephrine secretion during the arousal state [114]. Despite a clear correlation has not been demonstrated yet, data on anxious behavior after fragmented or deprived sleep, and manic-like behaviors that were induced in mice by sleep fragmentation, shed light on potential involvement in bipolar disorder [115, 116], which would be consistent with the expression of markers of astroglial damage [117] and AQP-4 altered gene expression in bipolar disorder individuals [98]. In light of a growing interest in this topic, we also found a preprint study on the correlation between ADHD and the glymphatic system [118] and an abstract on SCZ and the glymphatic system [119], that were not included in this review for methodological reasons. Nevertheless, it is extremely important to outline and consider the limitations of the present study: several of the included studies included data obtained from a mouse model, and few studies yet are available on human samples, moreover, not all studies employed the same methodology for assessment nor the same markers, finally, many of the included studies were conducted in a cross-sectional manner, and thus could not address a direct causal relationship between impairment of the glymphatic system and psychiatric disorders. The lack of longitudinal studies, especially on subjects with a formal psychiatric diagnosis, stands out as a major limitation in obtaining specific and clarifying data, as it is crucial to consider the difficulty in determining whether glymphatic system impairments should be considered as consequences of psychiatric disease, or the impairment of such system could be actively participating in the pathogenesis of psychiatric pathologies.

## CONCLUSION

In conclusion, the present review aims to highlight the role of the glymphatic system in the context of psychiatric disorders, while underlining the need for implementation of research on this topic, as well as for markers definition and adaptation of specific radio diagnostic tools. Future studies are needed to replicate on human samples all psychiatry-related glymphatic research carried out on murine models. Moreover, it seems of utmost importance to take into consideration homogeneous samples of patients, with a formal psychiatric diagnosis, or specific patient subgroups, within a longitudinal assessment to yield better information and implement our knowledge on this topic.

## Figures and Tables

**Fig. (2):**
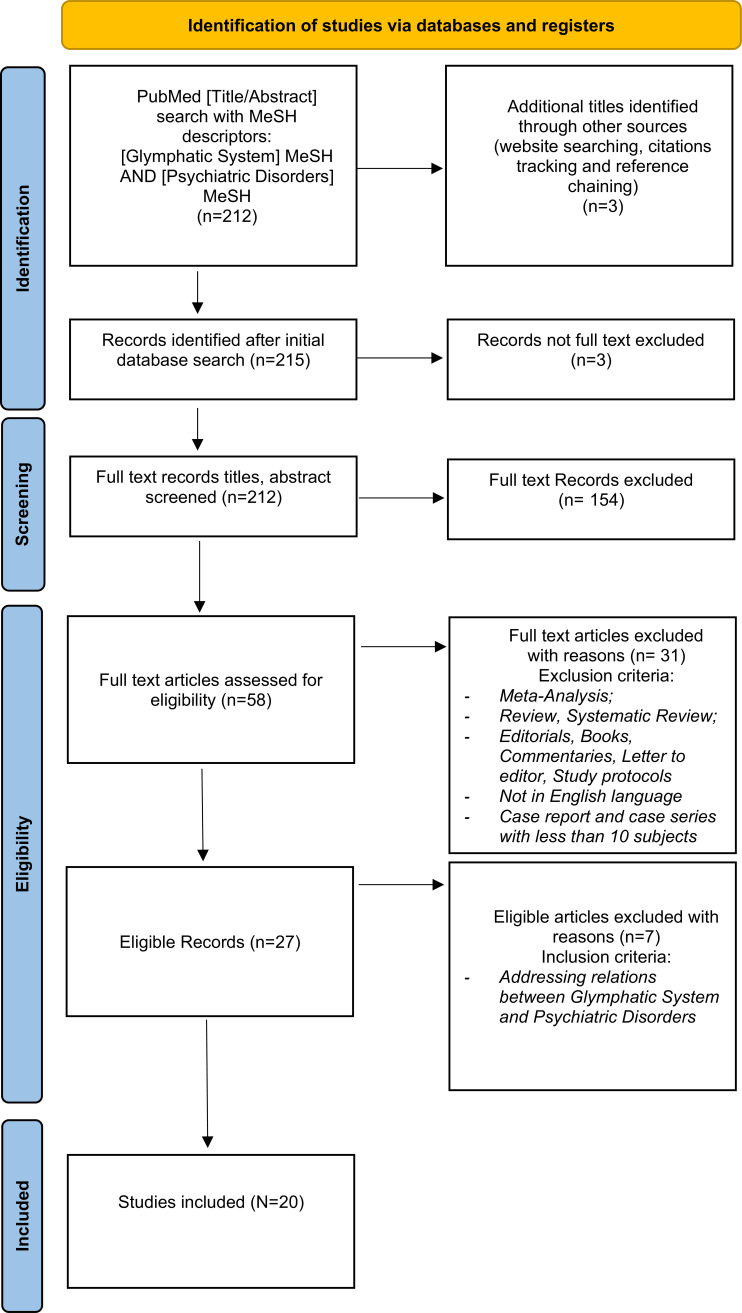
PRISMA 2020 flow diagram of included studies.

**Table 1:** Glymphatic system and psychiatric disorders characteristics of the studies included in the review.

**Characteristics of Studies Assessing the Relation between the Glymphatic System and Psychiatric Disorders (n = 20)**																									
**Authors, Year/** **References**	**Title**	**Location of Study**	**Sample Size**	**Psychiatric Disorder**	**Aim of the Study**	**Principal Findings**	**Implications**
Achariyar *et al.*, 2016 [40]	Glymphatic distribution of CSF-derived apoE into brain is isoform specific and suppressed during sleep deprivation	USA	Male C57BL/6 J and NG2-DsRed (Tg(Cspg4-Ds Red.T1)1Akik/J) mice.	Sleep Disorders	To establish whether apoE in CSF, secreted by the choroid plexus, is distributed into the brain, and whether this distribution pattern was altered by sleep deprivation.	The glymphatic fluid transporting system contributes to the delivery of choroid plexus/CSF-derived human apoE to neurons.	Sleep deprivation suppresses glymphatic CSF-derived apoE distribution into the brain and its clearance.
Wu *et al.*, 2020 [62]	Polymorphisms in the Human Aquaporin 4 Gene Are Associated with Schizophrenia in the Southern Chinese Han Population: A Case-Control Study	Taiwan	292 patients with Schizophrenia and 100 healthy controls.	Schizophrenia	To study the relationship of AQP4 gene polymorphisms and Schizophrenia (SCZ).	Three SNPs were found. In comparison to healthy controls, patients had higher T-allele frequencies at rs1058424 and G-allele frequencies at rs3763043. Furthermore, there is an association between the decreased risk of SCZ and the AA genotype at both rs1058424. The TCG haplotype was associated with a potential risk of SCZ, while the ACA haplotype was associated with a decreased risk of SCZ and retained statistical significance after Bonferroni correction.	An etiological reference for schizophrenia is provided by the association between AQP4 gene polymorphisms and schizophrenia in the Southern Han Chinese population.
Wu *et al.*, 2018 [63]	Human Aquaporin 4 Gene Polymorphisms and Haplotypes Are Associated With Serum S100B Level and Negative Symptoms of Schizophrenia in a Southern Chinese Han Population	Taiwan	190 patients with Schizophrenia	Schizophrenia	To investigate whether the gene polymorphisms and haplotype of AQP4 are associated with serum S100 calcium-binding protein B (S100B) level and clinical symptoms in patients with schizophrenia (SCZ).	Patients with the TAA haplotype of the AQP4 polymorphism are likely to have increased serum S100B level, negative symptoms and poor control of neuroinflammation. A logS100B level >1.78 may be sufficiently specific to predict a higher severity of negative symptoms.	AQP4 seems to influence brain neuroinflammation in SCZ because of its important role in maintaining BBB integrity, structure, and permeability. This study provides possible association between the involvement of genetic variations in the AQP4 gene and the functional outcome of patients with schizophrenia.
Li *et al.*, 2022 [64]	Children with autism spectrum disorder present glymphatic system dysfunction evidenced by diffusion tensor imaging along the perivascular space	China	30 children with Autism Spectrum Disorder and 25 healthy controls in this study.	Autism Spectrum Disorder	To assess glymphatic system function in autism spectrum disorder (ASD) compared to healthy controls through diffusion tensor imaging (DTI) along the perivascular space (DTI-ALPS).	The DTI-ALPS index was strongly and positively associated with age. In patients with ASD, there is a glymphatic system dysfunction. This is intimately correlated to age.	In patients with ASD, there is a glymphatic system dysfunction. This is intimately correlated to age. This study suggests the importance of the DTI-ALPS approach in assessing the function of the glymphatic system in ASD.
Liu *et al.*, 2020 [65]	Polyunsaturated fatty acid supplement alleviates depression-incident cognitive dysfunction by protecting the cerebrovascular and glymphatic systems	China	Adult male C57BL/6 mice.	Depression	To test the prediction that the chronic unpredictable mild stress (CUMS) manifests in disturbed glymphatic function and that dietary supplementation with Polyunsaturated fatty acid (PUFA) could improve these deficits while alleviating the depression-associated cognitive decline.	PUFA supplementation rescued depression-like behaviors of CUMS mice, reduced neuroinflammation and cerebrovascular dysfunction, ultimately improved cognitive performance, all of which accompanied by restoring glymphatic system function.	The CUMS depression model entails suppression of the glymphatic system. PUFA supplementation rescued most behavioral signs of depression and the associated cognitive dysfunction by restoring the underlying glymphatic system disruption and protecting cerebral vascular function.
Xia *et al.*, 2017 [66]	Mechanism of depression as a risk factor in the development of Alzheimer's disease: the function of AQP4 and the glymphatic system	China	Male Mice C57BL/6 and FVB/NTg (GFAP-GFP) 14Mes/J.	Depression	To identify the pathogenetic relationship between depressive disorder and Alzheimer’s disease (AD) through a chronic unpredictable mild stress (CUMS) model to determine the function of the glymphatic pathway by using fluorescence tracers. Immunohistochemistry was used to assess the accumulation of endogenous mouse and exogenous human amyloid beta 42 (Aβ42) in CUMS-treated mice with or without treatment with antidepressant fluoxetine.	Glymphatic pathway circulation was impaired in mice treated with CUMS; moreover, glymphatic pathway dysfunction suppressed Aβ42 metabolism, because the accumulation of endogenous and exogenous Aβ42 was increased in the brains of the CUMS-treated mice. However, treatment with fluoxetine reversed these destructive effects of CUMS on the glymphatic system. In anhedonic mice, the expression of the water channel aquaporin 4 (AQP4), a factor in glymphatic pathway dysfunction, was down-regulated in cortex and hippocampus.	The dysfunction of the glymphatic system suggested why a history of depression may be a strong risk factor for AD in anhedonic mice.
Ranti *et al.*, 2022 [67]	Perivascular spaces as a marker of psychological trauma in depression: A 7-Tesla MRI study	USA	21 patients with Major Depressive Disorder and 27 healthy controls.	Depression/ Trauma related-disorders	Investigated the link between glymphatic neuroanatomy *via* perivas-cular spaces (PVS) and trauma experience in patients with major depressive disorder (MDD) and in healthy controls using 7-Tesla MRI and a semi-automated segmentation algorithm.	The number of traumatic events was correlated with total PVS volume in MDD patients and the overall population. The number of traumatic events eliciting horror was positively correlated with total PVS volume in MDD patients and the overall population. Age correlated positively with PVS count, PVS total volume, and PVS density in all participants.	Results suggest a relationship between glymphatic dysfunction related to blood brain barrier (BBB) integrity and psychological trauma, and that glymphatic impairment may play a role in trauma-related symptomatology.
Chen *et al.*, 2021 [68]	Cerebral small vessel disease may worsen motor function, cognition, and mood in Parkinson's disease	China	421 patients with Parkinson disease.	Depression/ Anxiety	To explore the multi-dimensional effects of Cerebral Small Vessel Disease (CSVD) on Parkinson’s Disease (PD) outcomes (motor, cognition, and depression/anxiety).	Comorbid CSVD may contribute to multi-dimensional dysfunction in patients with PD and different CSVD imaging markers may play distinct roles in PD outcomes. The management of cerebral vascular disease may be crucial to maintain motor and non-motor functions in PD.	The total CSVD burden was significantly associated with depression/anxiety in patients with PD.
Liu *et al.*, 2017 [69]	Continuous theta burst stimulation facilitates the clearance efficiency of the glymphatic pathway in a mouse model of sleep deprivation	China	108 males C57BL/6 mice.	Sleep disorders/ Anxiety	To explore the effect of continuous theta burst stimulation (cTBS) with a two-photon *in vivo* imaging on glymphatic pathway clearance in normal and SD C57BL/6J mice. Aquaporin-4 (AQP4) polarization was detected by immunofluorescence. Anxiety-like behaviors were measured using open field tests.	SD reduced influx efficiency along the peri-vascular space (PVS), disturbed AQP4 polarization and induced anxiety-like behaviors. Continuous theta burst stimulation (cTBS) significantly attenuated the decrease in efficiency of solute clearance usually incurred with SD, restored the loss of AQP4 polarization and improved anxiety-like behavior in SD animals.	Results implied that cTBS had the potential to protect against neuronal dysfunction, including anxiety-like-behavior, induced by sleep disorders.
Vasciaveo *et al.*, 2023 [70]	Sleep fragmentation affects glymphatic system through the different expression of AQP4 in wild type and 5xFAD mouse models	Italy	Two-months-old no carrier male mice (control mice) and 2- and 6-month-old male B6SJL-Tg (APPSwFlLon, PSEN1 ∗ M146L ∗ L286V) 6799Vas/Mmjax (5xFAD) mice.	Sleep Disorders/ Anxiety	To analyze the effect of sleep fragmentation in wild type and 5xFAD mouse models. Anxiety-like behaviors were measured using open field tests.	Fisher's multiple comparison test revealed increased anxiety in fragmented (F) 5xFAD mice by reducing the time spent in the open arms compared to not fragmented (NF) 5xFAD and F-wt mice, which in contrast spent more time in the open arms compared to their control group (NF-wt). Sleep fragmentation induces a general acceleration of AD progression in 5xFAD mice, while in wild type mice it affects cognitive behaviors in particular learning and memory.	Aquaporin-4 (AQP4) modulation is a crucial player of the glymphatic system activity. In particular, sleep fragmentation differentially affects aquaporin-4 channel (AQP4) expression according to the stage of the disease, with an up-regulation in younger animals, while such change cannot be detected in older ones. After sleep fragmentation, 5xFAD mice manifested an increase in anxious and hyperactive behavior.
Zhang *et al.*, 2020 [71]	Aquaporin 4 deletion exacerbates brain impairments in a mouse model of chronic sleep disruption	China/ USA	Adult AQP4 null mice and wild-type (WT) mice.	Sleep Disorders	To investigate pathophysiological roles of astroglial aquaporin 4 (AQP4), a functional regulator of glymphatic clearance, in a mouse model of chronic sleep disruption (SD).	Aquaporin 4 deletion resulted in an impairment of glymphatic transport and accumulation of β‐amyloid and Tau proteins in the brain following SD. AQP4 null SD mice exhibited severe activation of microglia, neuroinflammation, and synaptic protein loss in the hippocampus, as well as decreased working memory, compared with WT-SD mice.	AQP4-zmediated glymphatic clearance ameliorates brain impairments caused by abnormal accumulation of metabolic wastes following chronic SD, thus serving as a potential target for sleep‐related disorders.
Siow *et al.*, 2022 [72]	Association of Sleep, Neuropsychological Performance, and Gray Matter Volume with Glymphatic Function in Community-Dwelling Older Adults	China	84 participants aged 60 years or older.	Sleep Disorders	To explore the associations between human glymphatic function, sleep, neuropsychological performance, and cerebral gray matter volumes.	N2 sleep duration and the apnea-hypopnea index were independently associated with DTI-ALPS. Higher DTI-ALPS was associated with better ECog language scores and better CERAD-NB word list learning delayed recall subtest scores after covarying for age and education. Higher DTI-ALPS was also associated with higher gray matter volume after controlling for age, sex, and total intracranial volume.	Significant associations were identified between glymphatic function and sleep, stressing the importance of sleep for brain health. This study also revealed associations between DTI-ALPS, neuropsychological performance, and cerebral gray matter volumes, suggesting the potential of DTI-ALPS as a biomarker for cognitive disorders.
Wang *et al.*, 2022 [73]	MRI-visible enlarged perivascular spaces: imaging marker to predict cognitive impairment in older chronic insomnia patients	China	156 patients with chronic insomnia and 79 age-matched healthy individuals.	Sleep Disorders	To explore whether MRI-visible enlarged perivascular spaces (EPVS) could be imaging markers to predict cognitive impairment in chronic insomnia patients.	MRI-visible EPVS in the frontal cortex, centrum semiovale, basal ganglia, and hippocampus of chronic insomnia patients with impaired cognition (ICG) significantly increased than that in normal cognition (NCG) patients. The increased MRI-visible EPVS in the frontal cortex, centrum semiovale, and basal ganglia were also associated with the increased CSF Aβ42, t-tau, and p-tau level in ICG patients. MRI-visible EPVS in the basal ganglia and centrum semiovale had high sensitivity and specificity in distinguishing ICG chronic insomnia patients from those with NCG.	MRI-visible EPVS in the basal ganglia and centrum semiovale might be valuable imaging markers to predict cognitive impairment in chronic insomnia patients.
Rainey-Smith *et al.*, 2018 [74]	Genetic variation in Aquaporin-4 moderates the relationship between sleep and brain Aβ-amyloid burden	Australia	462 aged 60 years or older.	Sleep Disorders	To determine whether genetic variation within AQP4 moderated the relationship between PSQI-derived self-reported sleep quality and brain Aβ burden as assessed by PET in cognitively normal older adults of the AIBL study.	One AQP4 variant, rs72878776, was associated with poorer overall sleep quality, while several SNPs moderated the effect of sleep latency (rs491148, rs9951307, rs7135406, rs3875089, rs151246) and duration (rs72878776, rs491148 and rs2339214) on brain Aβ-amyloid burden.	This study suggests that genetic variation of AQP4 moderates the relationship between sleep parameters and brain Aβ burden.
Piantino *et al.*, 2021 [75]	Link between Mild Traumatic Brain Injury, Poor Sleep, and Magnetic Resonance Imaging: Visible Perivascular Spaces in Veterans	USA	56 Iraq/ Afghanistan Veterans.	Sleep Disorders	To determine the relationship between the number of mild traumatic brain injury (mTBI), poor sleep, and PVS burden in a cohort of Iraq/ Afghanistan military veterans after adjusting for clinically relevant covariates and to establish the relationship between PVS burden and persistent post-concussive symptoms in this cohort.	The number of mTBIs sustained in the military is associated with PVS burden, and this relationship is modulated by poor sleep. In this cohort, increased PVS burden is associated with worse post concussive symptoms, particularly poor balance.	Quality of sleep modulates the relationship between the number of mTBIs sustained in the military and PVS volume. In persons with poor sleep, the increase in PVS volume observed with each subsequent mTBI is higher than in those who sleep well.
Shokri-Kojori, *et al.* 2018 [76]	β-Amyloid accumulation in the human brain after one night of sleep deprivation	USA	20 healthy controls tested after a night of rested sleep, referred to as rested-wakefulness (RW).	Sleep Disorders	To assess the effect of one-night SD on brain ABB with PET-FBB in healthy controls and to compare the measures to baseline brain ABB captured at the same time of the day but following a night of rested sleep [referred to as rested-wakefulness (RW)]. To replicate the previously reported association between sleep history and brain ABB (when measured after RW).	One night of sleep deprivation, relative to baseline, resulted in a significant increase in Aβ burden in the right hippocampus and thalamus. These increases were associated with mood worsening following sleep deprivation, but were not related to the genetic risk (APOE genotype) for Alzheimer’s disease. Baseline brain Aβ burden (ABB) in a range of subcortical regions and the precuneus was inversely associated with reported night sleep hours.	There is an association between SD-related increases in ABB and mood worsening supporting the functional significance of elevated ABB.
Eide *et al.*, 2021 [77]	Cerebrospinal fluid egress to human parasagittal dura and the impact of sleep deprivation	Norway	One group of individuals (n = 7) underwent one night’s total sleep deprivation while a control group (n = 9) was allowed unrestricted sleep.	Sleep Disorders	To examine whether CSF tracer enhancement in human parasagittal dura is affected by sleep deprivation.	After one night of sleep deprivation (at 24 h), there were neither evidence for altered tracer enrichment in the parasagittal dura, nor after a day of unrestricted sleep (at 48 h).	The hypothesis of altered molecular egress to parasagittal dura after sleep deprivation was not supported by this study.
Chen *et al.*, 2020 [78]	Cocaine-induced structural and functional impairments of the glymphatic pathway in mice	China	Adult male C57BL/6J mice.	Cocaine use disorder	To evaluate glymphatic function, including CSF-ISF exchange and solute clearance from the brain, using a mouse model of noncontingent cocaine exposure, during repeated cocaine exposure and after withdrawal.	Cocaine exposure, substantially impaired glymphatic function including CSF-ISF exchange and solute clearance from the brain.	Cocaine exposure impairs clearance of waste from the brain, which may contribute to the development of neurocognitive disorders in patients with drug addictions.
Lundgaard *et al.*, 2018 [79]	Beneficial effects of low alcohol exposure, but adverse effects of high alcohol intake on glymphatic function	USA	Awake, behaving C57Bl6 mice.	Alcohol use disorder	To investigate the effects of acute and chronic ethanol exposure and withdrawal from chronic ethanol exposure on glymphatic function.	Acute and chronic exposure to 1.5 g/kg (binge level) ethanol dramatically suppressed glymphatic function in awake mice. Chronic exposure to 1.5 g/kg ethanol increased GFAP expression and induced mislocation of the astrocyte-specific water channel aquaporin 4 (AQP4), but decreased the levels of several cytokines. Glymphatic function increased in mice treated with 0.5 g/kg (low dose) ethanol following acute exposure, as well as after one month of chronic exposure. Low doses of chronic ethanol intake were associated with a significant decrease in GFAP expression, with little change in the cytokine profile compared with the saline group.	Ethanol has a J-shaped effect on the glymphatic system whereby low doses of ethanol increase glymphatic function. Conversely, chronic 1.5 g/kg ethanol intake induced reactive gliosis and perturbed glymphatic function, which possibly may contribute to the higher risk of dementia observed in heavy drinkers.
Liu *et al.*, 2020 [80]	Experimental alcoholism primes structural and functional impairment of the glymphatic pathway	China	Adult male C57BL/6J mice (8أ12 weeks of age) and Aqp4-knockout mice on a C57BL/6.	Alcohol use disorder	To investigate the exact mechanisms by which acute and chronic binge alcohol exposure impairs the glymphatic function in the brain.	The elevated release of β-endorphin and reduced cerebrovascular pulsatility after acute alcohol administration may account for the impairment of the glymphatic function. Chronic moderate alcohol consumption led to pronounced activation of astrocytes and a widespread loss of perivascular AQP4 polarization in the brain, which results in an irreversible impairment of the glymphatic function.	The study suggests that impaired glymphatic functions and reduced parenchymal Aβ clearance found in both acute and chronic alcohol treatment may contribute to the development of cognitive decline and dementia in alcoholism.

## LIST OF ABBREVIATIONS

AD: Alzheimer's Disease
ADHD: Attention Deficit and Hyperactivity Disorder
AQP-4: Aquaporin-4
ASDs: Autism Spectrum Disorders
AUD: Alcohol Use Disorder
Aβ42: Amyloid Beta 42
BBB: Blood-brain Barrier
CERAD-NB: Consortium to Establish a Registry for Alzheimer's Disease Neuropsychological Battery
CNS: Central Nervous System
CP: Choroid Plexus
CP: Choroid Plexus
CSF: Cerebrospinal Fluid
CSVD: Cerebral Small Vessel Disease
cTBS: Continuous Theta Burst Stimulation
CUD: Cocaine Use Disorder
CUMS: Chronic Unpredictable Mild Stress
DTI: Diffusion Tensor Imaging
DTI-ALPS: Along The Perivascular Space
ES: Escitalopram
GFAP: Glial Fribrillar Acid Protein
HAMA: Hamilton Anxiety Rating Scale
HAMD: Hamilton Depression Rating Scale
HC: Healthy Controls
ICG: Impaired Cognition
ISF: Interstitial Fluid
MDD: Major Depressive Disorder
MeSH: Medical Subject Headings
MRI: Magnetic Resonance Imaging
MS: Multiple Sclerosis
mTBIs: Mild Traumatic Brain Injuries
NCG: Normal Cognition
NVU: Neurovascular Unit
OCD: Obsessive-compulsive Disorder
OF: Open Field
PD: Parkinson's Disease
PDs: Personality Disorders
PET: Positron Emission Tomography
PRISMA-ScR: Preferred Reporting Items for Systematic Reviews and Meta-Analyses extension for Scoping Reviews
PSQI: Pittsburg Sleep Quality Inventory
PTSD: Post-traumatic Stress Disorder
PUFAs: Polyunsaturated Fatty Acids
PVSs: Perivascular Spaces
ROS: Reactive Oxygen Species
RW: Rested-wakefulness
S100B: S100 Calcium-binding Protein B
SCZ: Schizophrenia
SDs: Sleep Disorders
SNPs: Single Nucleotide Polymorphisms
SUDs: Substance Abuse Disorders
USA: United States
VRS: Virchow Robin Space
WT: Wild‐type
β-EP: β-endorphin

## STANDARDS OF REPORTING

PRISMA guidelines and methodology were followed.
